# Grid cell firing properties vary as a function of theta phase locking preferences in the rat medial entorhinal cortex

**DOI:** 10.3389/fnsys.2014.00193

**Published:** 2014-10-14

**Authors:** Ehren L. Newman, Michael E. Hasselmo

**Affiliations:** Center for Memory and Brain, Department of Psychological and Brain Sciences, Boston University, Boston, MAUSA

**Keywords:** acetylcholine, navigation, spatial tuning, theta, medial entorhinal cortex, grid cells

## Abstract

Theta rhythmic fluctuations in the hippocampal–entorhinal circuit are believed to reflect rapid transitions between modes of mnemonic processing. Specifically, activity at the trough and peak of CA1 pyramidal layer theta is thought to correspond to retrieval and encoding related processing, respectively. Spatially tuned “grid cells” in layers II and III of the medial entorhinal cortex preferentially spike during the trough and peak phases of theta, respectively. Such differences suggest differential involvement of these layers to the processes of retrieval and encoding. It remains unknown, however, if the properties of grid cells that spike preferentially at the trough vs. the peak of theta differ systematically. Such putative differences would offer insights into the differential processing that occurs during these two phases. The goal of the present work was to contrast these types of grid cells. We found that significant functional dissociations do exist: trough locked grid cells carried more spatial information, had a higher degree of head direction tuning, and were more likely to phase precess. Thus, grid cells that activate during the putative retrieval phase of theta (trough) have a greater degree of location, orientation, and temporal tuning specificity relative to grid cells that activate during the putative encoding phase (peak), potentially reflecting the influence of the retrieved content. Additionally, trough locked grid cells had a lower average firing rate, were more likely to burst, and were less phase locked to high-gamma (∼80 Hz). Further analyses revealed they had different waveforms profiles and that systemic blockade of muscarinic acetylcholine receptors reduced the spatial tuning of both types, although these differences were only significant for the peak locked grid cells. These differences suggest that trough and peak locked grid cells are distinct populations of neurons.

## INTRODUCTION

The prominent 6–10 Hz theta rhythmic fluctuation in neural activity of the hippocampal–entorhinal circuit is believed to reflect rapid, frequent transitions between modes of neural information processing (Hasselmo et al., 2002; Norman et al., 2006; Douchamps et al., 2013). Compellingly, the neurons in each sub-region of the circuit fire at distinct phases of the local field theta rhythm, offering insights into the different types of processing that likely occur at each phase of theta (Dragoi and Buzsáki, 2006; Mizuseki et al., 2009). Entorhinal input, carrying information from cortical processing streams, for example, arrives in hippocampal area CA1 at the opposite phase of theta than does input from hippocampal auto-association region CA3 (Buzsáki et al., 1983; Brankack et al., 1993). This temporal segregation led to the idea that distinct mnemonic encoding and retrieval phases exist, corresponding to the arrival of the entorhinal and CA3 input, respectively (Hasselmo et al., 2002).

A reversal in theta phase locking preferences between layers II and III of the entorhinal cortex (Mizuseki et al., 2009) likewise suggests a differential involvement of these layers in these mnemonic processes. Consistent with this idea, the projections from these layers into the hippocampus are largely segregated. Layer III selectively projects to the distal dendrites of area CA1 (Steward, 1976). Layer II, on the other hand, projects predominantly to the dentate gyrus (DG) and area CA3 (Steward and Scoville, 1976; Amaral and Witter, 1989), but a subset of layer II neurons also synapse onto inhibitory interneurons among the distal dendrites of area CA1 (Kitamura et al., 2014).

Our understanding of what information is carried by these entorhinal inputs to the hippocampus has exploded in the last decade (Fyhn et al., 2004; Hafting et al., 2005; Sargolini et al., 2006; Solstad et al., 2008; Deshmukh and Knierim, 2011; Zhang et al., 2013). Of particular relevance, the input from the dorsal medial entorhinal cortex carries spatially tuned signals. Grid cells, observed in both layers II and III, fire at regular intervals spanning the surface of testing enclosures, marking the corners of a tessellating equilateral triangle pattern. The phase locking preferences of grid cells vary over layers of the entorhinal cortex. However, the relationship between those phase locking preferences and tuning properties of grid cells remains poorly characterized.

The goal of the work described here was to compare the spiking, tuning, and phase locking properties of grid cells of the medial entorhinal cortex as a function of the phase of theta on which they spike preferentially. We report that the spiking of grid cells locked to the trough theta in layer III of the medial entorhinal cortex (which is in phase CA1 pyramidal layer theta) carried more spatial information, had a higher degree head direction tuning, and were more likely to phase precess. Additionally, trough locked grid cells had a lower average firing rate, were more likely to burst, and were less phase locked to high-gamma (∼80 Hz) fluctuations in the local field potential. Analyses of the basic physiology of trough and peak locked grid cells revealed different profiles of the extracellularly recorded waveforms and showed that, while systemic blockade of muscarinic acetylcholine receptors reduced the spatial tuning of both types, these differences were only significant for the peak locked grid cells. These categorical differences further indicate that grid cells are not a single homogenous population. This data provides additional constraints on models of processing within the medial entorhinal cortex and between it and the hippocampus.

## MATERIALS AND METHODS

All animal procedures and surgery were conducted in strict accordance with National Institutes of Health and Boston University Animal Care and Use Committee guidelines.

### SUBJECTS

The data included in the analyses described here consisted of grid cells from nine male Long-Evans rats. All animals weighed 350–400 g at the time of surgery, were individually housed, were maintained at 90% of their free-feeding weight following their full recovery after surgery and maintained on a 12:12 h light–dark cycle. All procedures were conducted during the light cycle.

### SURGICAL PROCEDURE

For implantation of electrodes, rats were anesthetized with isoflurane and a ketamine/xylazine mixture, the skull surface was exposed, and five to nine anchor screws and one ground screw (located anterior and lateral to the bregma skull suture) were affixed to the skull. Tetrodes were constructed out of four 12.7 μm diameter nichrome wires twisted together that were gold plated to bring the impedance at 1 kHz down to 150–300 kΩ. Tetrodes were either bundled together and mounted in a single screw drive from Axona Ltd. or loaded into a multi-screw hyper-drive giving an average of 300 μm inter-tetrode spacing. The Axona Ltd. drive was implanted into the brain at 4.5 mm lateral, 0.3 mm anterior from the transverse sinus, 1.5 mm deep and were tilted 10^∘^ in the anterior direction. The tetrode bundle of the hyper-drive was centered on a point 4.5 mm lateral and was placed anterior to the transverse sinus so that the posterior edge of the bundle abutted the sinus. No angle was used when implanting the hyper-drives. The screws and drives were attached to the skull with dental acrylic. The rats recovered for 7 days before behavioral testing and recording began.

### BEHAVIORAL PROTOCOL

The spiking properties of neurons were recorded as the rats foraged in an open field enclosure or completed laps on a circle track as specified for each analysis. In the open field, animals foraged for cereal bits in a walled (50 cm high) rectangle enclosure (75 or 150 cm by 100 cm). In the circle track task, the animals received cereal bits for each lap completed in either direction (56 cm radius; 8 cm wide track). A black curtain surrounded both enclosures. The open field allowed for the identification of grid cells in all animals. A subset of rats was also run on the circle track. All circle track trials were performed under low light conditions in a curtained area without overt extra-enclosure visual cues.

### DATA ACQUISITION

Data collection was performed with the Axona Ltd. DacqUSB system. The same system tracked the position of a large and small LED on the recording head stage to track the position and head direction of the animal at a rate of 50 Hz. Signals recorded from the tetrodes were filtered and amplified to record local field potentials (bandpass 1–250 Hz; amplified ∼2,500X) and unit activity (bandpass 0.6–6.7 KHz; amplified ∼6–10,000X). Potential spike waveforms were identified by a rising slope that crossed a 65–100 μV threshold and stored to disk along with a 32-bit time stamp. Spiking activity of individual units was discriminated oﬄine using the Tint cluster cutting software (Axona Ltd., Hertz, UK). The polarity of unit activity recordings were reversed (i.e., recorded: reference – signal) so as to have the extracellularly recorded voltage deflection resulting from an action potential oriented upward; the local field potential referenced to directly to the ground screw without a reversal of polarity (i.e., recorded: signal – ground).

### DATA INCLUSION CRITERIA

The data included in the analyses described here were selected from a database of recordings collected by the authors in the course of conducting other experiments. Multiple recordings from the same cell were frequently available. A single recording was selected among those available for each cell based on the quality of the behavior and the recorded spiking activity. Specifically, sessions without good coverage or without sufficient stability in the waveform were disqualified. Cluster quality was quantified with the isolation distance and L ratio metrics (Schmitzer-Torbert et al., 2005). Only cells with an isolation distance of 10 or greater were included in the analyses described here. Among multiple sessions recorded for a single unit, the recording with the greatest isolation among the qualified sessions was then taken to be the representative recording. The isolation quality did not differ significantly between the populations of trough and peak locked cells (21.5, 22.8, respectively; *p* = 0.1, *W* = 8,001). Analyses of spiking behavior on the circle track were restricted to those cells confirmed to exhibit significant grid tuning in the open field enclosure. Analyses of the influence of muscarinic blockade on spiking were performed on a subset of the cells as were analyzed previously (Newman et al., 2014). Finally, to avoid potential confounds introduced by a theta phase reversal in layer II (Mitchell and Ranck, 1980), cells from tetrodes located beyond this phase reversal were removed from all analyses. To identify these recordings, the pattern of theta–gamma phase–amplitude coupling was examined for evidence of phase reversals as described elsewhere (Newman et al., 2013). By this approach, 18 cells were putatively recorded from beyond the theta phase reversal and were removed. None of the effects were qualitatively changed when these cells were removed. Because of this exclusion, all references to peak or trough locked indicate the peak or trough of the theta as is observed in layer III (which is in phase with CA1 pyramidal layer theta).

### SCOPOLAMINE ADMINISTRATION

Cholinergic modulation sensitivity analyses were performed by comparing data collected immediately prior to and following systemic muscarinic blockade was performed as described previously (Newman et al., 2014). Briefly, scopolamine hydrobromide (0.5 mg/kg) was administered intraperitoneally. Volume matched injections of sterile saline were administered as a control condition. Animals rested on a pedestal for 15 min following the injection to allow for the drug to take effect before running the post-injection trial. A minimum of three intervening days was allowed between sequential scopolamine injections.

### DATA ANALYSIS

**FIGURE 1:**
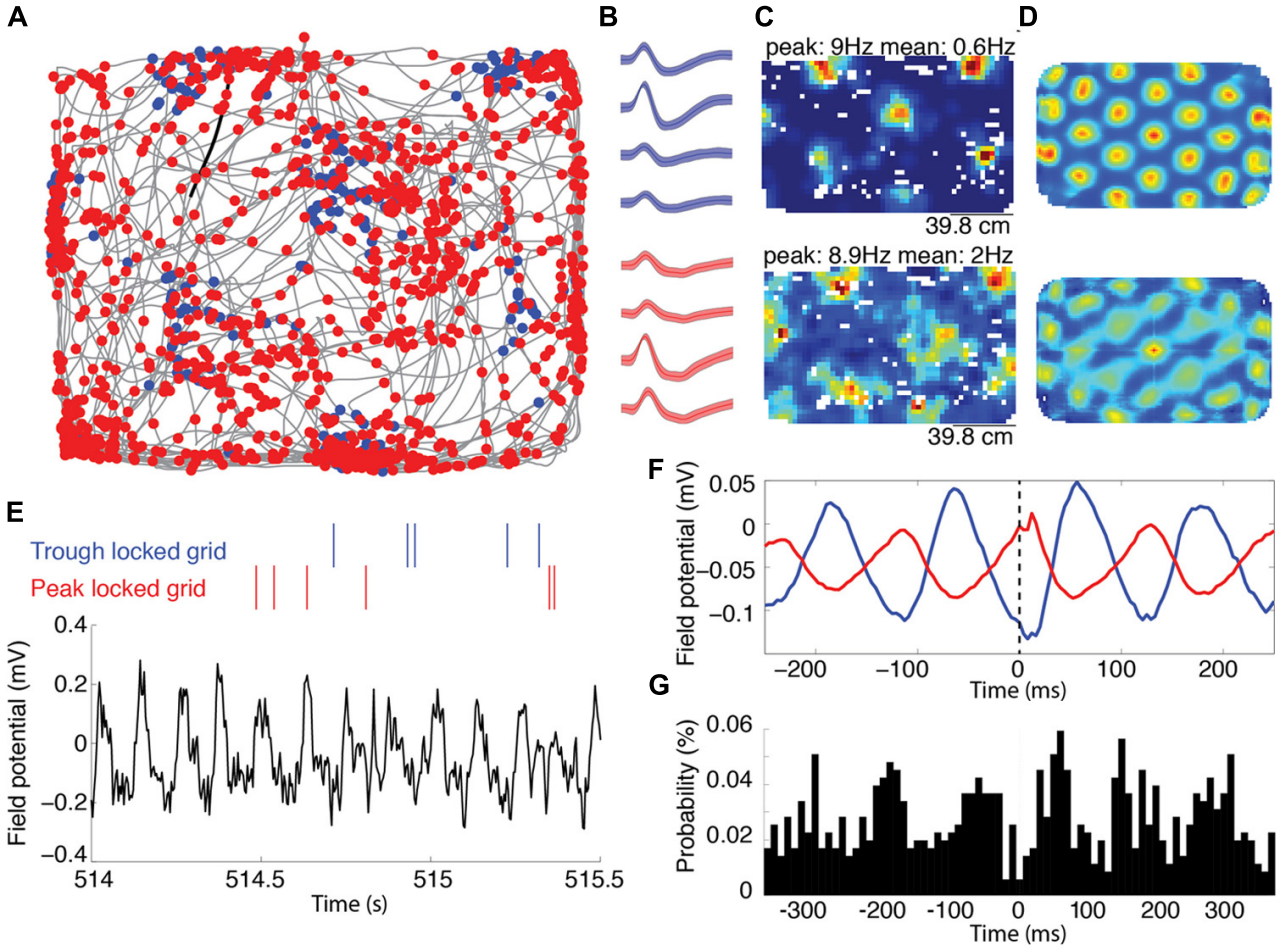
**Representative grid cells locked to the peak and trough of the local theta rhythm recorded on the same tetrode. (A)** Trajectory of a rat, shown in grey, foraging for food reward during an open field trial. The blue and red dots mark the location of the animal each time the trough or the peak locked grid cells spiked, respectively. **(B)** The average waveforms of the two cells shown in **(A)**. The blue and red waveforms correspond to the blue and red marks in **(A)**, respectively. The shading around each line reflects the standard error on the mean over waveforms. **(C)** Rate maps indicating the average spike rate (Hz) of the spatial firing of the trough and peak locked cells. The peak firing rate (corresponding to the deepest red) and average firing rate is indicated above each map. The top and bottom maps correspond to the blue and red cells indicated in **(A,B)**. **(D)** Two-dimensional autocorrelograms of the rate maps shown in **(C)**. **(E)** Representative alignment between the spike timing and the local field theta rhythm. The raw local field potential is shown in black with color-coded spike rasters plotted above. Spikes from the red cell occurred with high likelihood at the peaks of the ongoing theta, while spikes from the blue cell occurred with high likelihood at the trough of theta. **(F)** Spike triggered averages of the unfiltered local field potential for the two cells. These averages show a trough and peak at zero-lag (marked with a dashed line) for the blue and red cells, respectively. **(G)** A cross-correlation between the spike times of the trough and peak locked cells, showing peaks at about ±60 ms, corresponding to a half theta cycle lag.

The data analyses sought to evaluate the temporal and spatial spiking properties of individual grid cells and to characterize the differences between peak locked and trough locked grid cells (**Figure 1**). The properties selected for inclusion in the described analyses were those that offered the greatest insights into the heterogeneity of grid cells or those that offer insight into how sub-classes of grid cells relate to the network physiology. All analyses were performed in Matlab with scripts developed in-house unless specified otherwise.

#### Grid cell identification

Only those cells that exhibited significant grid tuning in an open field testing enclosure were included in the analyses described here. Significant grid tuning was assessed as described previously (Newman et al., 2014). In short, for each cell, the modified grid score (Brandon et al., 2011) was calculated from the 2D autocorrelogram of the rate map constructed with 3 cm pixels with 5 cm Gaussian smoothing. This grid score was then compared to a null distribution of grid scores, constructed by shifting the alignment of between spike times and animal position for 200 iterations, to identify cells for which the true grid score was greater than at least 95% of the scores computed in the permutation analysis (Domnisoru et al., 2013).

#### Phase locking

**FIGURE 2:**
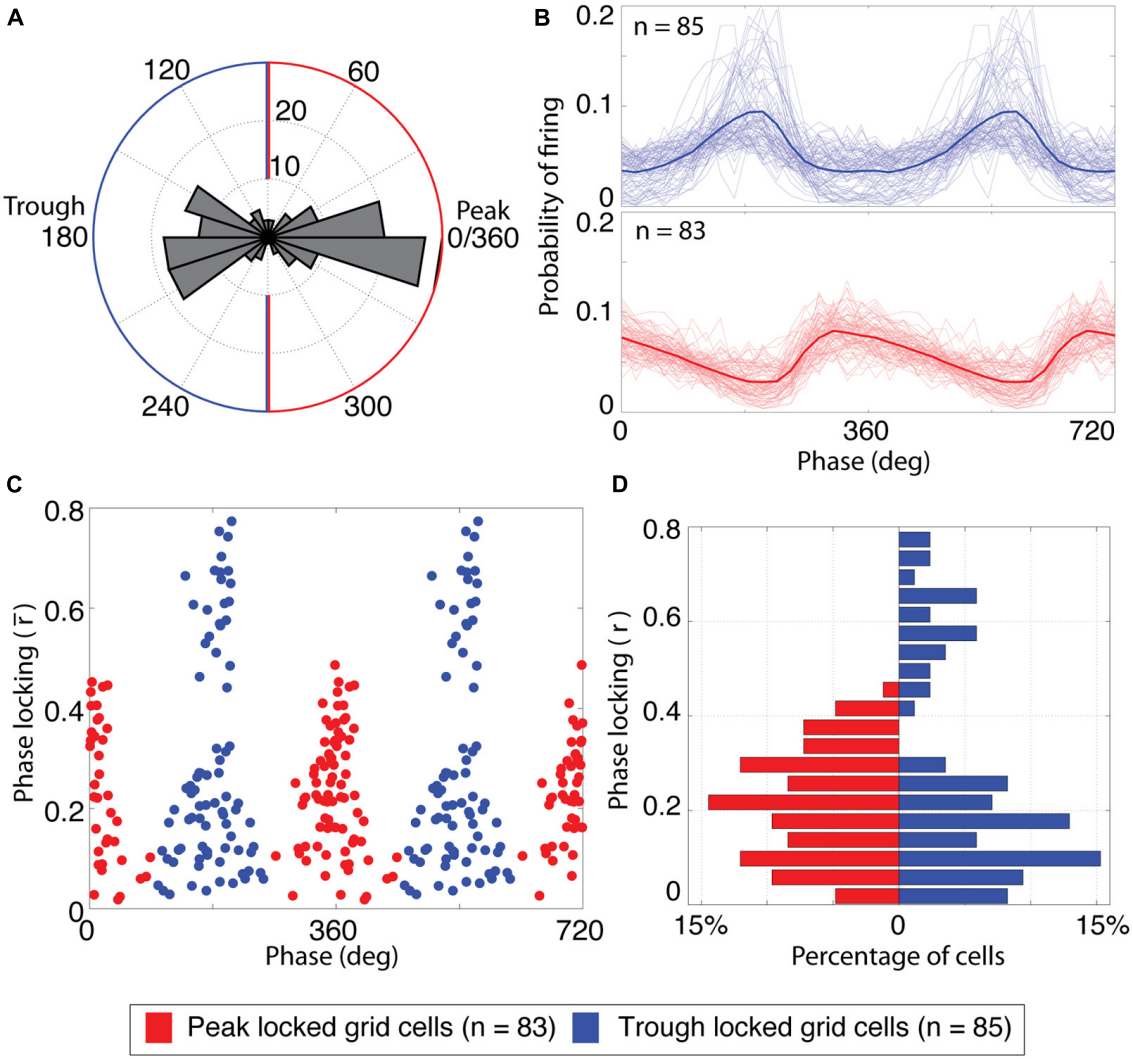
**Grid cells lock to either the peak or the trough of theta. (A)** Histogram of the preferred firing phase of theta for all cells with significant grid tuning (*n* = 168). The distribution was strongly bimodal, with most cells locking to either the peak or trough of theta. The blue and red outlines indicate the classification boundaries of the preferred firing phases that were classified as trough and peak locked grid cells, respectively. **(B)** Probability of firing over phases for trough locked (top) and peak locked cells (bottom). The mean over cells of a respective type is shown as a thick line, individual cells are shown as thin lines. **(C)** Phase locking properties of individual cells, plotted as a function of preferred firing phase and phase locking strength. Blue (red) dots reflect trough (peak) locked grid cells. The *x*-axis is repeated twice for improved clarity of the bimodality. **(D)** Histogram of phase locking strengths plotted separately for peak (red bars on left) and trough (blue bars on right) locked cells.

The phase at which individual cells were most likely to fire was a robust predictor of how those cells were likely to score on other metrics. To compute phase locking, the phase of theta in the local field potential at which each spike occurred was computed using the Hilbert transform of a bandpass filtered signal (6–10 Hz; butterworth filter; order 4). A mean resulting vector was then calculated from N unit-length vectors with phase angles equal to the theta phase angles to which each of the N spikes from a cell was aligned. The length of the vector reflected the degree of phase locking and the angle reflected the phase of preferred firing. Phase locking histograms, such as shown in **Figure 2**, were built for illustration purposes with 36 10^∘^ bins. Additional analyses of phase locking, in the gamma bands for example, were performed in a modified fashion. Phase locking was computed for each of 96 logarithmically spaced frequency bands spanning from 10 to 123 Hz and the time varying phase each was estimated using a Morlet wavelet of width 6, consistent with previously used methods (Newman et al., 2013). The degree of phase locking and preferred phase was then quantified as the mean resultant length computed over all of the spike phases for each frequency band.

#### Categorization of grid cells as peak vs. trough locked

A scatter plot of the preferred theta phase of firing vs. the mean resulting length described above for all grid cells showed a striking bimodal distribution with peaks at 0 and 180^∘^ and local minima around 90 and 270^∘^ as shown in **Figure 2C**. This bimodal distribution was thus split into peak locked grid cells (those for which the preferred phase of firing was closer to 0^∘^ than to 180^∘^) and trough locked cells (with a preferred phase closer to 180^∘^ than 0^∘^).

These phase locking preferences were calculated in reference to the local field potential, that is, the field potential recorded from the same tetrode on which each cell was isolated. Because recordings from beyond the theta phase reversal that occurs in layer II, near the border of layer I, were excluded (see Data Inclusion Criteria) the theta phases examined would be expected to be in phase with CA1 pyramidal layer theta (Mizuseki et al., 2009).

#### Head direction tuning

**FIGURE 3:**
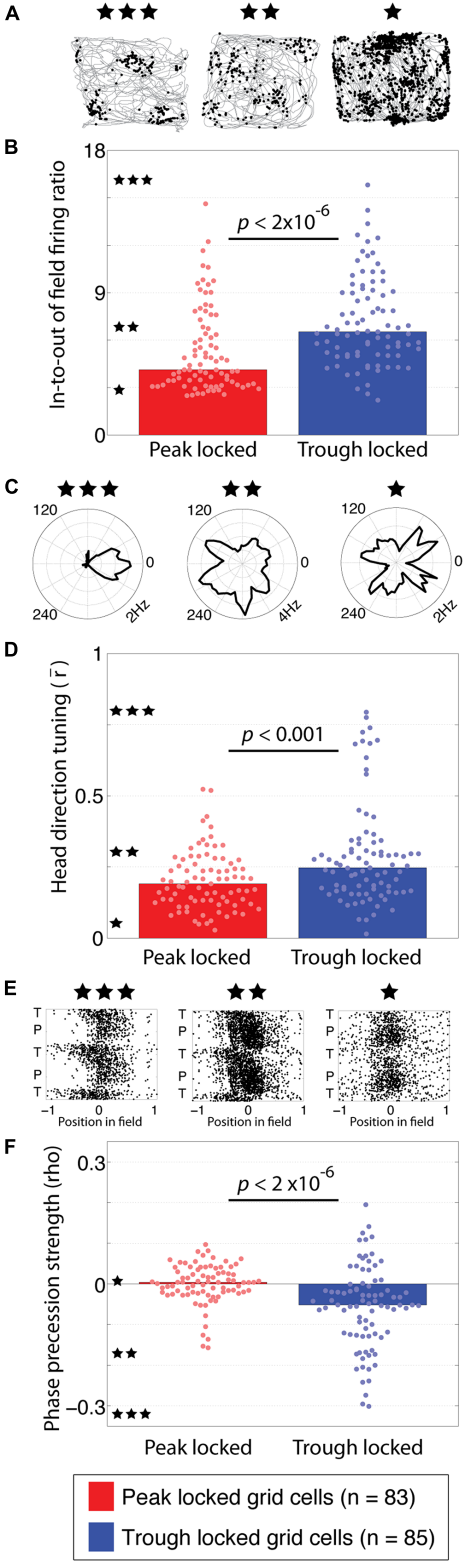
**Functional tuning differences between peak and trough locked grid cells. (A)** Trajectory plots of three grid cells illustrating the range of spatial information observed in the grid cell spiking. The left example, marked with three stars, reflects the highest spatial information observed. The middle (two stars) and right-most (one star) examples reflect the modal spatial information carried by the trough and peak locked grid cells, respectively. **(B)** Distributions of in-to-out of field firing ratios in peak and trough locked grid cells, indicating that trough locked grid cells have a greater in-to-out of field firing ratio on average. The stars located along the *y*-axis indicate the in-to-out of field firing ratio of the examples with the corresponding number of stars shown in **(A)**. **(C)** Head direction tuning of three grid cells, illustrating the range of tuning observed, from strongest to weakest moving from left to right. The black line marks the average firing rate of the respective cell when the animal faced each direction. **(D)** Distributions of head direction tuning, quantified as the length of the mean resultant vector, indicating that trough locked grid cells have a greater degree of head direction tuning on average. **(E)** Spiking of three grid cells plotted as a function of position in field and theta phase, illustrating the range of phase precession strengths observed, from most to least robust moving from left to right. **(F)** Distributions of phase precession strength, quantified as the rho value resulting from the linear–circular correlation between position in field and theta phase, indicating that trough locked grid cells exhibit stronger phase precession on average. The bar indicating the median of the peak locked cells on the left is plotted but is indistinguishable from zero. In all panels, data from trough (peak) locked cells are plotted in blue (red), the bars indicate the medians of the respective distributions and the dots mark the values observed for individual cells.

Quantification of the strength of head direction tuning for each cell was performed by computing the length of a mean resultant vector calculated from N unit vectors with phase angles equal to the head direction angle of the animal at the time of each of the N observed spikes. This vector varied in length from 0, for no net head direction tuning, to 1, for perfect head direction tuning (see examples shown in **Figure 3C**). Secondary analyses were performed on the subset of cells with robust head direction tuning. To be included among the cell in these analyses, cells must exhibit significant tuning as assessed via a Watson’s *U*2 test computed on the distribution of head directions at the time spikes were observed, as done previously (Newman et al., 2014).

#### Phase precession

For each cell, the strength of phase precession for each cell was quantified as the rho value obtained from a linear–circular correlation, described in full elsewhere (Kempter et al., 2012), between the linear position of the animal in the field at the time of each spike and the phase of theta at the time of each spike (see examples shown in **Figure 3E**). The position of the animal within the firing field in the open field was calculated as described elsewhere (Climer et al., 2013). In short, a time varying estimate of the expected firing rate was calculated by comparing the trajectory to the whole-session rate map and a Hilbert transform was used to numerically assess the progress through bouts of increased expected firing rate. Cells were identified as exhibiting significant phase precession if the correlation analysis resulted in a *p*-value less than 0.05 and the derived rho value was negative.

#### Theta rhythmicity

Beyond phase locking and phase precession, we also assessed the extent to which the spike time autocorrelogram exhibited theta rhythmicity. That is, if the autocorrelogram contained a prominent rhythmic peak at a lag consistent with theta modulation of the spiking. Rhythmicity was calculated using the depth of modulation metric described elsewhere (Sharp and Koester, 2008; Newman et al., 2014). In short, the depth of modulation was calculated as the difference in the number of spikes occurring at first trough of the autocorrelogram and the first non-zero-centered peak, divided by the number of spikes in the first non-zero-centered peak.

#### Quality of spatial tuning

We used several approaches to quantify various aspects of the spatial tuning. These included: *spatial information* and *in-to-out of field firing ratio* to quantify the contrast between in-field and out-of-field firing; the *grid score* to quantify the spatial periodicity of the tuning pattern; *rate map stability* to quantify the similarity of the spatial tuning between the first and second half of a testing trial; and *spatial coherence* to quantify the smoothness of the spatial tuning. In the case of circle track enclosures, the *1D grid cell classification rate* served to classify each cell as a grid cell or not according to a list of qualifying criteria. In each metric, higher values reflect greater spatial tuning quality. Each metric is described in turn.

The contrast of the firing fields from background activity was assessed using the *spatial information* and *in-to-out of field firing ratio* metrics. *Spatial information* was computed as described previously (Skaggs et al., 1993). The *in-to-out of field firing ratio* computed the ratio between rate map pixels with high firing rate and those with low firing rate. Pixels were separated into high and low firing rates bins using a threshold defined as 33% of the peak firing rate in the map. The ratio between the mean firing rate of all high firing rate pixels and the mean of all low firing rate pixels was then used as a quantification of the contrast. Note, the percentile of the maximum that we used as the threshold was chosen to loosely correspond to a 1 SD radius on an idealized Gaussian distribution of spikes in a field. However, the same qualitative pattern of results was obtained irrespective of the precise threshold we tested (10, 33, and 50%).

The spatial periodicity of the observed tuning was quantified differently for open field and circle track trials. To analyze 2D open field rate maps, the modified grid score [as described by (Brandon et al., 2011)] was used. To analyze 1D circle track rate maps, the spatial periodicity score was used, as described elsewhere (Newman et al., 2014).

The stability of the underlying spatial tuning was quantified using the *rate map stability* score by correlating the rate maps derived from the first and second half of the testing trial as described by (Newman et al., 2014). For circle track rate maps, the stability score was computed for rate maps computed from clockwise laps and counter clockwise laps separately and then linearly combined based on the amount of time spent running in each direction.

The smoothness of the rate map was quantified with the *spatial coherence* metric as described elsewhere (Kubie et al., 1990). In the present study, spatial coherence was calculated as the correlation between the rate map and a variant of the rate map where the activity in every pixel is replaced by the mean of the adjacent pixels. Importantly, this was performed on non-smoothed rate maps. In the case of circle track rate maps, the spatial coherence was computed for rate maps computed from clockwise laps and counter clockwise laps separately and then linearly combined based on the amount of time spent running in each direction.

Finally, for circle track enclosure trials, the 1D grid cell classifier (Domnisoru et al., 2013) was used to assess the number of cells that meet a set of criteria previously proposed for detecting tuning in a 1D testing environment that is predictive of a cell with grid tuning in 2D testing environments. Readers should refer to Domnisoru et al. (2013) for a full set of specifications.

#### Waveform analysis

**FIGURE 4:**
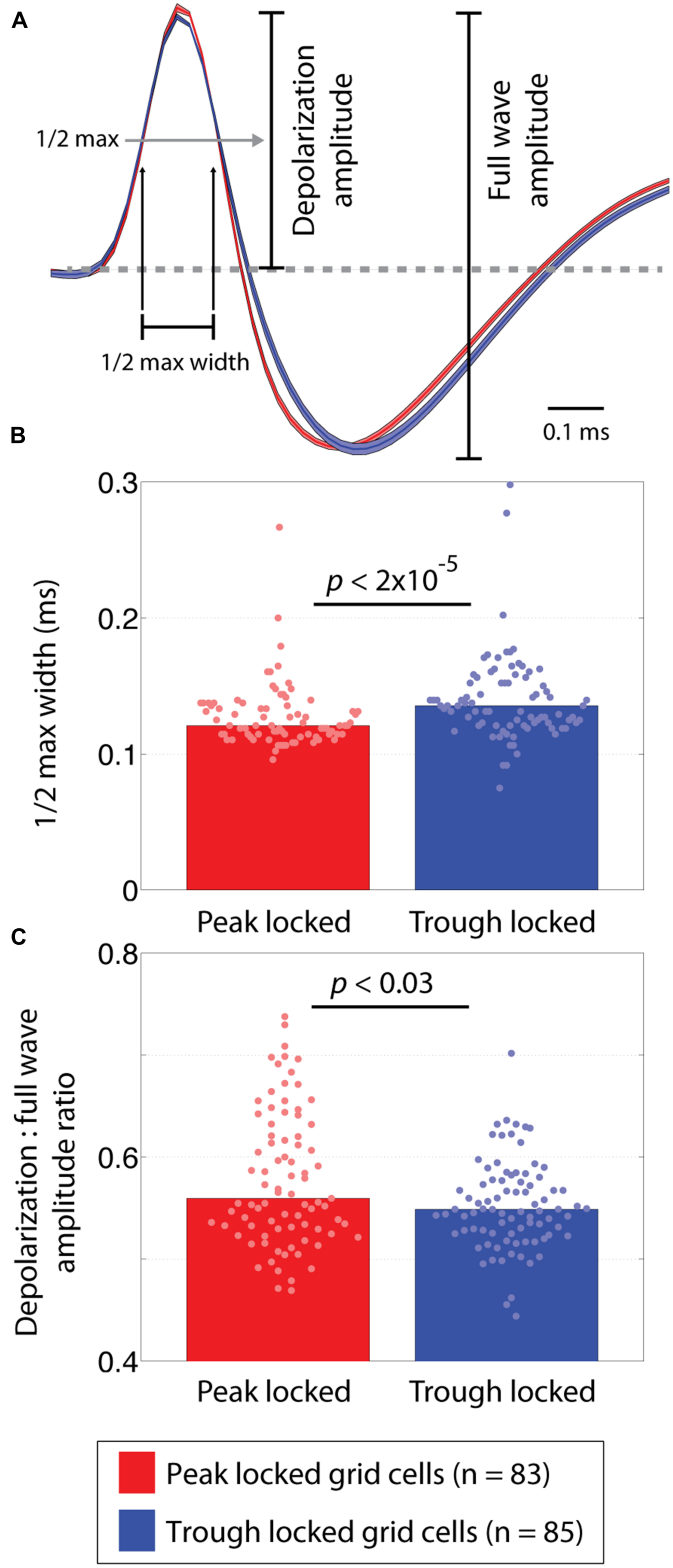
**Waveform differences between peak and trough locked grid cells. (A)** Average waveforms of trough and peak locked cells shown in blue and red, respectively. Shading around each line reflects the SE on the amplitude-normalized waveforms. The annotations indicate how the depolarization phase and full wave amplitudes were defined as well as how the half-max width of the depolarization phase was defined. **(B)** The distribution of half-max widths in peak and trough locked grid cells, indicating that the waveforms of trough locked grid cells had wider depolarization phases. **(C)** The distributions of the depolarization amplitude to the full wave amplitude ratios, indicating that trough locked grid cells had a shorter relative depolarization phase. In all panels, data from trough (peak) locked cells are plotted in blue (red), the bars indicate the medians of the respective distributions and the dots mark the values observed for individual cells.

To compare the shape of the waveforms, a number of features were considered. The first of which was the width of the half max of the depolarization phase of the waveform. This was computed as the amount of time between when the rising and falling edges of the depolarization peak crossed a threshold defined as 50% of the maximum voltage of the peak. Similarly, the width of the half minimum of the hyperpolarization phase was computed as the time between when the falling and rising edges of the hyperpolarization crossed a threshold defined as 50% of the minimum voltage of the hyperpolarization. To decrease aliasing in the widths, the waveform was linearly interpolated from 48 samples up to 480 samples. This allowed the estimated widths to vary in steps of a 10th of a sample instead of in integer steps. The third feature assessed the ratio between amplitude of the depolarization relative to the full waveform. These metrics are illustrated in **Figure 4A**.

### STATISTICS

Statistical comparisons focused on assessing the difference between peak locked and trough locked grid cells over a variety of metrics. For each metric, we tested if the distribution of samples differed significantly from a Normal distribution with a Kolmogorov–Smirnov test. In all cases, we found that we could reject the null hypothesis that our data was sampled from a normal distribution. As such, all statistical comparisons of independent samples were performed using the non-parametric Wilcoxon rank sum test and comparisons of paired samples were performed using the non-parametric Wilcoxon signed-rank test. Accordingly, the test statistic reported for tests of independent samples is W, the summed rank, and the test statistic reported for tests of paired samples is *T*, the signed rank. Analyses comparing the number of observed cells counts relative to the expected number of cell counts used a Chi-Square test. For all tests, the null hypothesis was rejected if the observed *p*-value was less than or equal to 0.05. Consistent with the use of non-parametric tests, all reported summary statistics reflect the medians of the distributions.

## RESULTS

The goal of the analyses described here was to test if the spiking properties of peak locked and trough locked grid cells differed in a significant fashion. As described in detail below, we found that grid cells that lock to the peak and trough of layer III theta (which is in phase with CA1 pyramidal layer theta) differed significantly with respect to the quality of spatial tuning, the degree of head direction tuning expressed, and the incidence of phase precession. The two types of grid cells also differed with respect to average firing rate, likelihood to burst, and the strength of phase locking to the local high-gamma rhythm (∼80 Hz). Analyses of the extracellularly recorded waveforms also revealed significant differences. Finally, while systemic blockade of muscarinic acetylcholine receptors reduced the spatial tuning of both types, these differences were only significant for the peak locked grid cells. Each of these results is described in detail below.

### GRID CELLS LOCK TO THE PEAK OR TROUGH OF THETA

Among the 168 cells exhibiting significant grid-like spatial tuning recorded from the medial entorhinal cortex of rats, individual cells preferentially spiked to either the peak or the trough of theta. **Figure 1A** shows the spatial distribution of spiking for two representative grid cells recorded from the same tetrode in the same trial (waveforms of these cells are shown in **Figure 1B**). The rate maps and 2D autocorrelograms of these rate maps illustrate the characteristic grid tuning pattern, with fields of increased spiking occurring at regularly spaced intervals, marking the apexes of a grid of equilateral triangles (**Figures 1C,D**). Notably, these two cells fired on opposite phases of the 6–10 Hz theta rhythm of the local field potential. **Figure 1E** shows a representative trace of the unfiltered local field potential as well as rasters indicating when the cells spiked relative to the prominent theta rhythm. These rasters show that the blue cell spiked near the troughs of theta and that the red cell spiked near the peaks of theta. The spike triggered averages of the local field potential from the entire testing trial for these two cells were 180^∘^ out of phase, demonstrating the reliability of these phase locking preferences (**Figure 1F**). Accordingly, the spike time cross-correlation between these two cells (shown in **Figure 1G**) had a trough at a lag of 0 ms and peaks at a lags of about ±60 ms, indicating that the cells typically fired with a minimum of a half theta cycle lag of each other.

Analysis of the phase locking preferences of all 168 grid cells recorded in open field testing enclosures revealed a striking bi-modal distribution of preferred firing phases as shown in **Figure 2A**. As was observed in the representative cells shown in **Figure 1**, grid cells were locked either to the trough of theta (phase of 180^∘^, *n* = 85) or just prior to the peak of theta (0 or 360^∘^, *n* = 83). To compare the firing properties of the cells that lock to the trough vs. the peak, each cell was labeled as either trough or peak locked as a function of whether the phase of preferred spiking for that cell was closer to 180 or 360^∘^. Phase locking histograms (i.e., the probability of observing spikes at each phase of theta) for both types of grid cell are shown in **Figure 2B**. These histograms illustrate that the trough locked grid cells (top plot) have a roughly symmetric probability of firing around the phase of maximal firing on average whereas the peak locked cells (bottom plot) have a skewed probability of firing around the peak with a long tailing edge. Among the trough locked cells, however, the profile of the phase locking histogram was less consistent than that observed across peak locked cells, visible as greater variability among the light blue lines in the top panel of **Figure 2B**. This variability is also apparent when the strength of phase locking is plotted as a function of the phase of preferred firing, as shown in **Figure 2C**. This figure indicates that trough locked grid cells (plotted in blue) show a bimodal distribution of phase locking strength whereas the peak locked grid cells (plotted in red) show a unimodal distribution of values (see also **Figure 2D**).

### PHASE LOCKING PREDICTS FUNCTIONAL TUNING PROPERTIES

Our goal in pursuing these analyses was to establish whether grid cells differed systematically as a function of their phase locking preferences. Toward this end, we analyzed the degree of grid tuning, the intra-trial tuning stability, the spatial coherence of the tuning, the spatial information of the tuning, the degree of head direction tuning, and the strength of phase precession. In short, trough and peak locked grid cells were matched with regard to the degree of grid tuning, tuning stability, and spatial coherence. They differed significantly, however, with respect to the degree of spatial information of the tuning and the likelihood to exhibit head direction tuning or phase precession. Each is described in turn.

The first question was whether trough and peak locked grid cells differed with respect to the nature of their spatial tuning. When compared with the grid score, however, the trough and peak locked grid cells were not significantly different (1.02, 0.88, respectively; *p* = 0.69, *W* = 6,886). The tuning of both trough and peak locked grids were also equivalently stable between the first and second half of individual testing trials (0.46, 0.41, respectively; *p* = 0.15, *W* = 6,556). The coherence of the unsmoothed rate maps was also similarly high for trough and peak locked grid cells (0.70, 0.68, respectively; *p* = 0.49, *W* = 6,793). These data are not shown in figure form.

When spatial tuning was assessed in terms of spatial information, however, a clear and robust difference emerged between the two groups of cells. Specifically, the tuning of the trough locked grid cells carried significantly greater spatial information than the peak locked cells (0.40 vs. 0.26 bits/spike; *p* < 0.001, *W* = 5,968). In other words, the distribution of firing rates over pixels of the rate map of the trough locked cells was significantly further from a uniform distribution than that of the peak locked cells. This difference can be seen in the rate maps of the representative cells shown in **Figure 1C**.

This difference in spatial information results from a greater level of out-of-field firing in the peak locked grid cells. This was quantified by computing the ratio of in-field firing to out-of-field firing for each rate map. Trajectory plots, showing the spiking of three different grid cells recorded with varying in-to-out of field firing ratios, are shown in **Figure 3A**. The left-most example shows the highest in-to-out of field firing ratio observed, with a value of 16.75, indicating that the average firing rate in-field was 16.75 times greater than the average firing rate out-of-field. The middle and left-most examples reflect the modal ratios observed among the trough and peak locked grid cells, respectively. Each is labeled with three, two, or one star and the corresponding ratio values are indicated along the *y*-axis of **Figure 3B** with a corresponding number of stars. The in-to-out of field firing ratio was significantly greater for the trough locked grid cells than for the peak locked grid cells (6.53, 4.13, respectively; *p* < 2 × 10^-6^, *W* = 5,476).

The next analyses sought to identify the alignment between theta phase locking preferences and head direction tuning. Previous work has shown that only a sub-set of grid cells exhibit robust head direction tuning (Sargolini et al., 2006). The head direction tuning of three different grid cells with varying degrees of tuning are shown in **Figure 3C**. Of the 168 grid cells analyzed here, 13 exhibited significant head direction tuning (i.e., Watson’s *U*2 of five or greater). Of those 13 cells with conjunctive grid/head direction tuning, 11 locked to the trough of theta. This distribution was significantly different from what would be expected by chance (χ^2^ = 5.14, *p* = 0.023). When considered as a population, the head direction tuning of the trough locked grid cells was significantly greater than that of the peak locked grid cells (0.25, 0.19, respectively; *p* < 0.0008, *W* = 5,952). Even after removing all of the conjunctive cells, the trough locked cells still had significantly greater head direction tuning relative to the peak locked cells (0.23, 0.18, respectively; *p* = 0.018, *W* = 6,433). This can be seen in the full distribution of head direction tuning values shown in **Figure 3D** even when discounting the long tail of higher head direction tuning values.

Another prominent feature of grid cell firing is phase precession, wherein a cell fires at progressively earlier phases of theta as the animal traverses through a firing field. As with head direction tuning, only a sub-set of grid cells have been found to exhibit significant phase precession (Hafting et al., 2008). Example scattergrams of spiking, plotted as a function of position in a firing field and theta phase, are shown in **Figure 3E** with the left-most example showing the strongest phase precession observed and the middle and right-most examples showing progressively less phase precession. The degree of phase precession exhibited by a cell was quantified using the rho value resulting from a correlation between position in field and phase of firing (Kempter et al., 2012). Of the trough locked grid cells 34% (25/85) exhibited significant phase precession, in contrast, 6% (5/83) of the peak locked grid cells exhibited phase precession (Climer et al., 2013). As a population, trough locked grid cells showed greater phase precession than the peak locked grid cells overall as shown in **Figure 3F** (-0.05, -0.0003, respectively; *p* = 1.3 × 10^-6^, *W* = 8,540).

In brief summary, among the 85 trough locked grid cells, 11 had significant head direction tuning (14%), 25 exhibited significant phase precession (34%), and 25 showed particularly high phase locking (rbar > 0.4; 29%). A series of secondary analyses were then used to assess the degree of overlap between these three sets of sub-populations. Notably, of the 11 trough locked conjunctive cells, 10 were strongly phase locked (91%) which was significantly more than would be expected by chance (χ^2^ = 19.7, *p* < 9 × 10^-6^) suggesting that there was a strong coincidence between head direction tuning and phase locking. Among the 83 peak locked grid cells, two were conjunctive grid cells (2%), eight were strongly phase locked (10%), and one of the conjunctive grid cells was also strongly phase locked.

None of the other combinations of phase locking, phase precession, and head direction occurred with a probability significantly above or below what would be expected by chance. Numerically, among trough locked grid cells, 6 of 11 conjunctive grid cells exhibited phase precession, and 6 of 25 strongly phase locked grid cells exhibited phase precession (χ^2^ = 2.58 *p* = 0.11) and (χ^2^ = 0.5 *p* = 0.48), respectively.

### PHASE LOCKING PREDICTS OTHER BASIC SPIKING PROPERTIES

The differences in functional firing properties between the peak and trough locked grid cells described above raised the question of whether these sub-groups may represent distinct physiological classes of neuron. To explore this hypothesis, basic firing properties of the sub-groups were compared, including the shape of the spike waveform, the average firing rate, the tendency to generate bursts of spikes, and the strength of phase locking to other frequency bands of the local field potential. All four metrics differed significantly between the cell types such that trough locked cells had a wider, but shorter, depolarizing phase of the extracellularly recorded waveform, had a lower average firing rate, were more likely to burst, and exhibited less phase locking to gamma rhythms. Each result is described in turn.

A number of features of the extracellularly recorded waveform were compared between the peak and trough locked cells (shown in **Figure 4A**). These included the width of the depolarization phase of the waveform, the width of the hyperpolarization phase of the waveform, and the ratio between the amplitudes of the depolarization phase and the full waveform. These analyses revealed that the waveforms differed subtly but significantly. Specifically, the width of the depolarization phase (at 50% of the maximum height) in trough locked grid cells was significantly greater than that in peak locked cells (*p* < 1.6 × 10^-5^, *W* = 5,654) as shown in **Figure 4B**. The widths of the hyperpolarization phase (at 50% of the maximum depth) did not differ (*p* = 0.54, *W* = 7,206). The ratio between the amplitude of the depolarization phase and the full waveform also differed significantly between the groups of grid cells (*p* < 0.03, *W* = 7,702) such that the depolarization was shorter in trough locked grid cells as shown in **Figure 4C**. This indicates that, despite having a longer lasting depolarization phase, the amplitude of the depolarization phase was relatively smaller than those of the peak locked grid cells. While the observed differences in waveform could result from differences in cluster quality between the groups, we found no significant differences between quality of the clusters when assessed in terms of isolation distance or the L ratio (*p* = 0.09, *W* = 7,543 and *p* = 0.19, *W* = 6,596, respectively; Schmitzer-Torbert et al., 2005).

**FIGURE 5:**
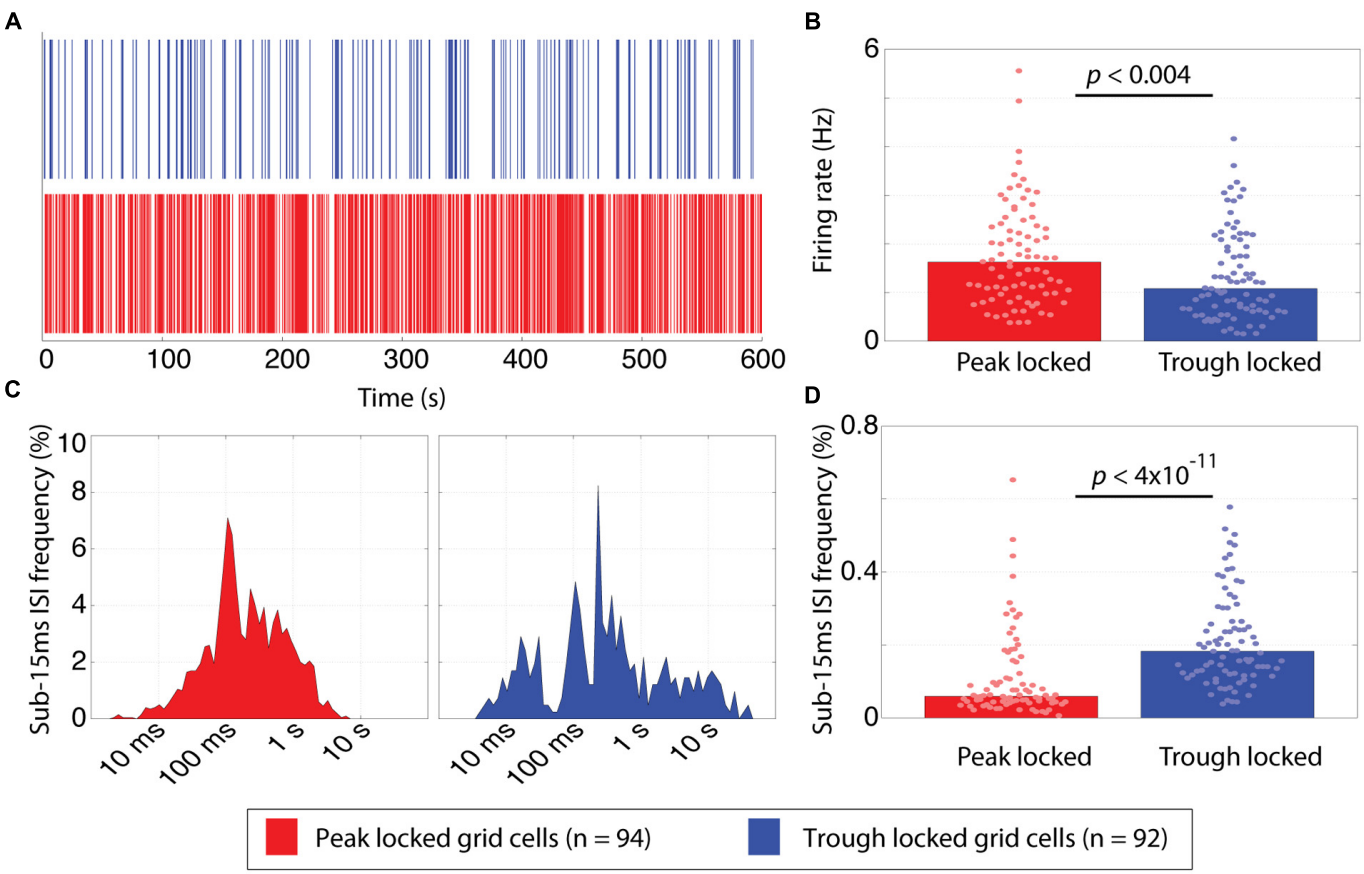
**Temporal firing differences between peak and trough locked grid cells. (A)** Spike rasters of representative trough (blue) and peak (red) locked grid cells, illustrating that the temporal firing pattern of the trough locked grid cells is sparser. **(B)** The distribution of mean firing rates of peak and trough locked grid cells, indicating that trough locked grid cells have a lower mean firing rate on average. **(C)** Inter-spike interval (ISI) histograms of the trough and peak locked cells shown in **(A)** showing few low latency ISIs for the peak locked cell (left) and an isolated cluster of low latency ISI for the trough locked cell (right). **(D)** The distribution of the frequency that sub-15 ms ISIs are observed in peak and trough locked grid cells, indicating that trough locked grid cells have a higher likelihood of exhibiting such short ISIs. In all panels, data from trough (peak) locked cells are plotted in blue (red), the bars indicate the medians of the respective distributions and the dots mark the values observed for individual cells.

Average firing rate and bursting also revealed significant differences between the trough and peak locked grid cells (**Figure 5**). With regard to firing rate, the median firing rate of the trough locked grid cells was significantly lower than that of the peak locked grid cells (1.08 Hz vs. 1.63 Hz; *p* < 0.004, *W* = 7,934) as shown in **Figure 5B**. With regard to bursting, the percentage of inter-spike intervals (ISIs) that were 15 ms or less was significantly greater in spike trains of the trough locked grid cells relative to the peak locked grid cells (18%, 6%, respectively; *p* < 4 × 10^-11^, *W* = 4,918) as shown in **Figure 5D**. In other words, the trough locked cells not only fired fewer spikes in total per unit time, but, when they did spike, they generate several spikes in rapid succession, such that, the temporal distribution of spiking was substantially more sparse than was seen in peak locked cells (visible in **Figure 5A** as more frequent white spaces between spikes).

**FIGURE 6:**
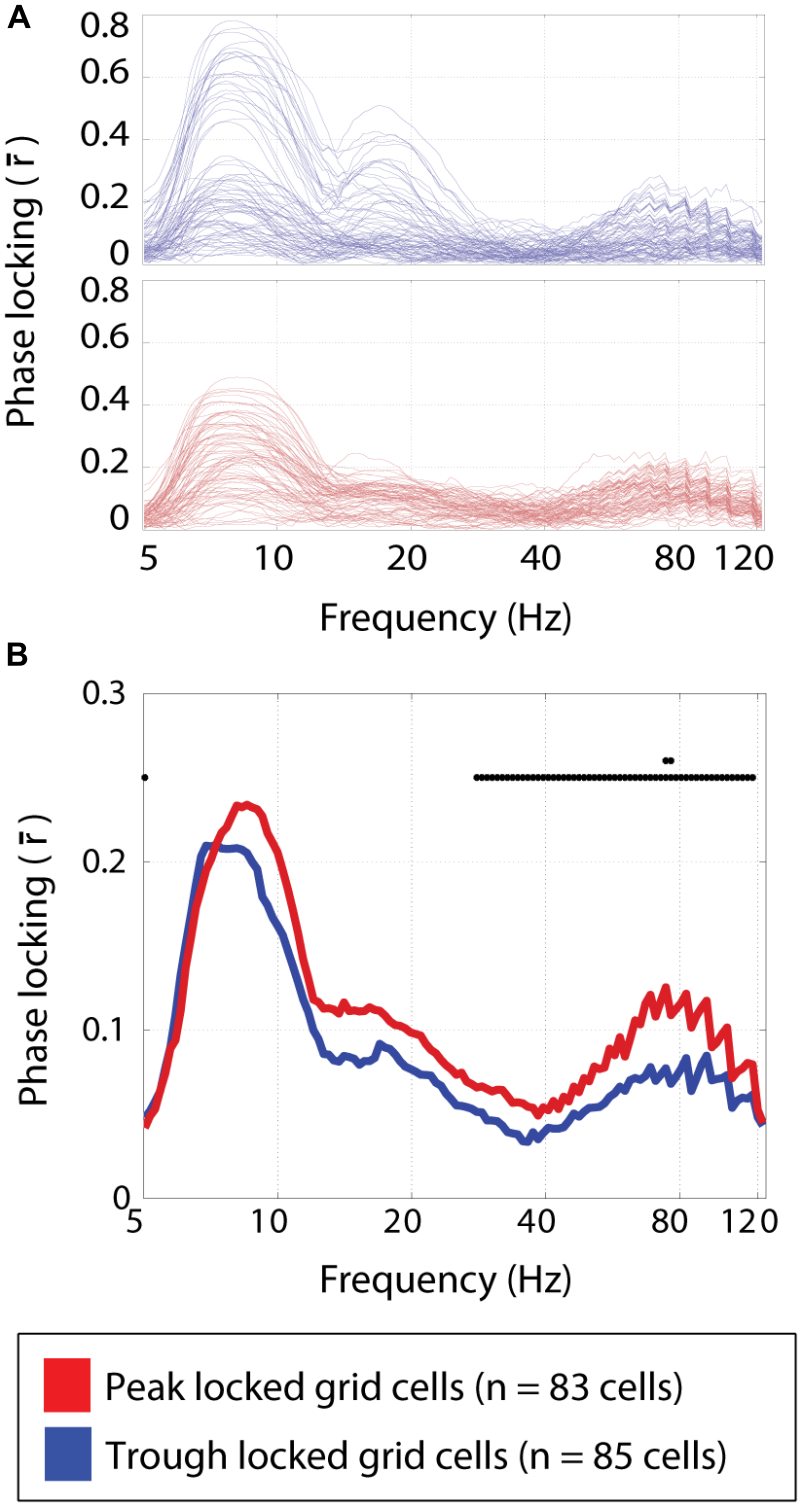
**Phase locking to frequency bands spanning 5–125 Hz. (A)** The strength of phase locking to frequencies spanning 5–125 Hz in the local field potential for individual trough locked (top) and peak locked (bottom) grid cells. Three local maxima can be seen, corresponding to theta (∼8 Hz) the harmonic of theta (∼16 Hz), and high gamma (∼80 Hz). **(B)** The median phase locking strength at each frequency for peak locked (red) and trough locked (blue) grid cells. Black dots plotted along the *y* = 0.25 line indicate significant differences between the two types of cells at *p* < 0.05, and the second row of dots indicate where the differences are significant a the *p* < 0.005 level, indicating a significant difference in the degree of phase locking in the high gamma band.

Multiple theta modulated gamma bands have been shown to exist in the medial entorhinal cortex (Newman et al., 2013). In particular, the higher of these gamma bands (60–120 Hz with a peak around 80 Hz) was modulated by the phase of theta such that the maximum amplitude occurred in time with the peak of the local field theta. Here, the phase locking preferences of each cell were assessed over a wide range of frequency bands. A qualitatively similar profile of phase locking strengths was observed for both the peak and trough locked grid cells as shown in **Figure 6A**. Specifically, local maxima in the strength of phase locking were observed in the theta band, around the harmonic of theta and in the high gamma band (centered around 80 Hz) as shown in **Figure 6B**. However, the relative height of these maxima differed between cell types. Specifically, the peak locked grid cells exhibited significantly greater phase locking to the high gamma band (0.12 vs. 0.08; *p* < 3 × 10^-4^; *W* = 8,183).

### PHASE LOCKING PREDICTS SENSITIVITY TO MUSCARINIC BLOCKADE

It has been previously shown that systemic blockade of muscarinic acetylcholine receptors (via I.P. administration of 0.5 mg/kg scopolamine) reduces the spatial periodicity, rate map stability, spatial coherence, grid cell classification rate, and theta rhythmicity of grid cells recorded from the medial entorhinal cortex (Newman et al., 2014). It was also shown that muscarinic blockade differentially reduced the amplitude of gamma locked to the peak of theta, suggesting that it may differentially affect processing at the peak and trough of theta (Newman et al., 2013). The current demonstration that the phase locking preferences of individual grid cells was predictive of different functional and basic firing properties raised the question of whether trough and peak locked grid cells were differentially sensitive to the muscarinic antagonist.

To address this question, recordings of grid cell activity as rats completed laps on a circular track for reward before and after a systemic administration of the muscarinic antagonist scopolamine (0.5 mg/kg), described previously (Newman et al., 2014), were reanalyzed; first, to identify which grid cells were locked to the peak vs. the trough of theta; second, to test if the spatial tuning of the two types of grid cells were differently effected by the drug manipulation, and third, to test if theta rhythmicity was differentially effected.

**FIGURE 7:**
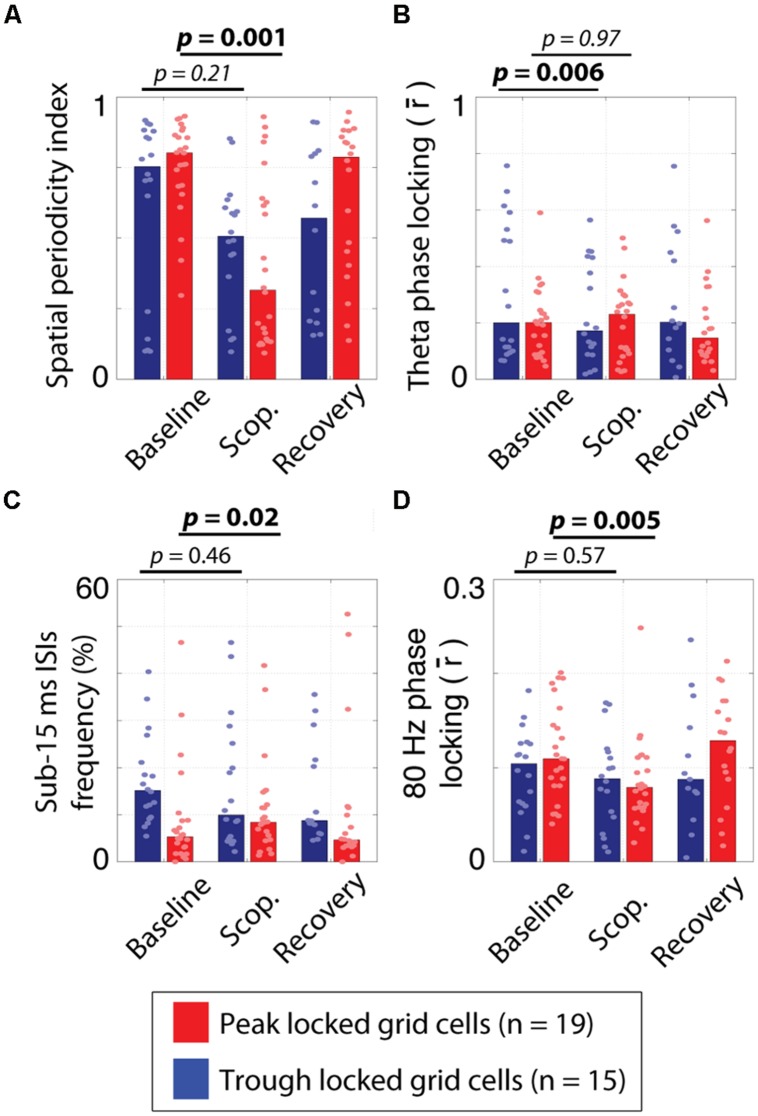
**Effects of systemic muscarinic acetylcholine receptor antagonism on peak and trough locked grid cells. (A)** The spatial periodicity index, indicating how periodic the spatial tuning on the circle track was during the pre-injection baseline trial, scopolamine injection trial (scop.) and post-injection recovery trial for the trough locked (blue bars) and peak locked (red bars) grid cells. While both cell types showed reductions, the effect was only significant for the peak locked grid cells. **(B)** The strength of phase locking to 6–10 Hz theta over trial and cell types. The theta phase locking was reduced in trough locked grid cells but not in peak locked grid cells. **(C)** The percentage of inter-spike intervals (ISIs) of 15 ms or less over trial and cell types. The frequency of occurrence of fasts ISIs increased in peak locked grid cells but not in trough locked grid cells. **(D)** The strength of phase locking to 80 Hz high gamma over trial and cell types. Gamma phase locking decreased in both cell types but was only statistically reliable in the peak locked grid cells. In all panels, the bar height reflects the median value and the dots mark the values observed for individual cells. Scop. = Scopolamine.

Of the 34 grid cells for which the influence of scopolamine was assessed, 19 locked to the peak of theta and 15 locked to the trough. The quality of the spatial tuning on the circle track was assessed with five metrics, with the goals of quantifying: (1) the *spatial information* of the firing in bits/spike; (2) the smoothness of the raw rate map (*spatial coherence*); (3) the *rate map stability* between the first and second half of each trial; (4) the periodicity of the tuning (*spatial periodicity index*); and (5) the *1D grid cell classification rate* as defined previously (Domnisoru et al., 2013). All five metrics were reduced by scopolamine in both types of grid cells. However, these effects were significant for the peak locked cells but only the reduction in rate map stability was significant for the trough locked cells. This difference in significance was likely partly due to the smaller n in the case of the trough locked cells (15 vs. 19) but was also likely to have been due to a difference in effect size between the groups. The median effect size was numerically larger for the peak locked cells for all five metrics and was significantly larger in the case of the spatial information and the 1D grid cell classification rate metrics. In short, while the spatial tuning of both the trough locked and peak locked grid cells was reduced by muscarinic blockade, peak locked cells showed a trend toward being more strongly impacted. The statistics corresponding to the contrasts mentioned here are shown in **Table 1** and the data from the spatial periodicity score are shown in **Figure 7A**.

**Table 1:** Statistical contrasts assessing the influence of a systemic administration of the muscarinic antagonist scopolamine on a variety of spatial tuning metrics (across rows) for both peak locked and trough locked grid cells (first two columns, respectively).

	Pre-injection vs. scopolamine injection Peak locked (*n* = 19)	Pre-injection vs. scopolamine injection Trough locked (*n* = 15)	Peak vs. trough
Spatial information (bits/spike)	0.15 vs. -0.06 ***p* < 0.0003**, *T* = 5	0.33 vs. 0.32 *p* = 0.65, *T* = 52	-0.19 vs. 0.01 ***p* < 0.02**, *W* = 330
Spatial coherence	0.55 vs. 0.45 ***p* = 0.04**, *T* = 44	0.54 vs. 0.45 *p* = 0.36, *T* = 44	-0.08 vs. -0.04 *p* = 0.47, *W* = 284
Rate map stability	0.60 vs. 0.49 ***p* < 0.04**, *T* = 43	0.68 vs. 0.52 ***p* < 0.047**, *T* = 25	-0.11 vs. -0.10 *p* = 0.65, *W* = 276
Spatial periodicity	0.80 vs. 0.32 ***p* < 0.001**, *T* = 13	0.73 vs. 0.49 *p* = 0.21, *T* = 38	-0.37 vs. -0.26 *p* = 0.15, *W* = 305
1D grid cell classification rate	79% vs. 5% ***p* < 0.0002**, *T* = 0	60% vs. 40% *p* = 0.26, *T* = 8	-74% vs. -20% ***p* < 0.02**, *W* = 325

*These contrasts show that scopolamine reduced the spatial tuning for both types of grid cells, it was only reliably statistically significant for the peak locked cells. A comparison between these effects across cell types (last column) revealed significantly larger effects were observed in peak locked grid cells for both spatial information and 1D grid cell classification rate.*

Previous analysis of the influence of muscarinic blockade on theta phase locking and theta rhythmicity in these cells resulted in non-significant trends toward drug induced reductions (Newman et al., 2014). Here, we found that, in trough locked cells, theta phase locking was significantly reduced (0.31 vs. 0.16; *p* < 0.006, *T* = 11), as was theta rhythmicity (0.52 vs. 0.36; *p* = 0.009, *T* = 14). In peak locked cells, however, we did not find significant changes to either theta phase locking (0.21 vs. 0.24; *p* = 0.97, *T* = 94) or theta rhythmicity (0.20 vs. 0.18; *p* = 0.31, *T* = 70). The difference in effect size between the trough and peak locked cells in the case of theta phase locking was significant (-0.10 vs. -0.01; *p* < 0.007, *W* = 184). The effects of scopolamine on theta phase locking are shown in **Figure 7B**. These results suggest that the weak statistical effects reported previously, were the consequence of the heterogeneity of grid cells.

In the course of reexamining this data set, we also sought to compare the influence of scopolamine on the other metrics we had already used to compare the peak and trough locked grid cells. Most notably, we found that scopolamine also had differential effects on the likelihood of bursting and gamma phase locking of the two types of grid cells. With regard to the likelihood of bursting, the percentage of ISIs of 15 ms or less increased significantly for peak locked grid cells (5% vs. 8%; *p* = 0.016, *T* = 35) and decreased non-significantly in trough locked grid cells (15% vs. 11%; *p* = 0.46, *T* = 47) as shown in **Figure 7C**. This difference between cell types trended toward significance (*p* = 0.066, *W* = 209). With regard to gamma phase locking, we observed a significant reduction in peak locked grid cells (0.11 vs. 0.08; *p* < 0.005, *T* = 24) and a non-significant increase in trough locked grid cells (0.11 vs. 0.09; *p* = 0.57, *T* = 50) as shown in **Figure 7D**.

## DISCUSSION

The trough and peak of CA1 pyramidal layer theta are believed to reflect distinct modes of mnemonic processing (Hasselmo et al., 2002; Norman et al., 2006). Here, we sought to test if the basic and functional firing properties of grid cells that fire preferentially on the trough vs. the peak of the local field theta rhythm (which is in phase with the CA1 pyramidal layer) differ in a systematic fashion thereby offering insights in to these mnemonic processes or into the grid cell network itself. We found a number of significant functional differences, wherein, relative to peak locked grid cells, trough locked grid cell spiking carried greater spatial information, was more likely to show significant head direction tuning, and to exhibit phase precession. The basic firing properties also differed such that, in trough locked grid cells, the average firing rate was lower, bursting occurred more often, and the depolarization phase of the waveform was shorter and wider than that observed in peak locked grid cells. Trough and peak locked grid cells also responded differently to systemic muscarinic acetylcholine receptor blockade. This was evident on metrics of spatial tuning, theta and gamma phase locking as well as bursting frequency. The differences described here suggest that trough and peak locked grid cells are distinct populations of neurons. Further, they show that the spiking of grid cells that activate during the putative retrieval phase of theta carries a greater degree of location, orientation, and temporal tuning specificity relative to grid cells that activate during the putative encoding phase, potentially reflecting the influence of the retrieved content. These results carry direct implications for physiological, computational, and theoretical models both regarding the relationship between mnemonic processing and the theta rhythm.

### PHENOTYPIC DIVERSITY BETWEEN PEAK AND TROUGH LOCKED GRID CELLS

The data described here demonstrate that peak and trough locked grid cells differ with respect to a variety functional and basic firing properties. Even within the peak and trough locked categories, we observed considerable heterogeneity in, for example, the strength of spatial or head direction tuning, the tendency to phase precess, or to exhibit strong phase locking. This phenotypic diversity is likely a reflection that a diverse range of cell types exhibit grid tuning. Within layers II and III of the medial entorhinal cortex, there are both stellate and pyramidal cells (Canto et al., 2008), both of which have been shown to exhibit grid tuning (Domnisoru et al., 2013). Intermediate variants of both the pyramidal and stellate cells exist, defined based on cytoarchitecture, demonstrating that substantial heterogeneity exists even within these populations (Canto and Witter, 2012). Determining the alignment between these subclasses of neuron to the phenotypes observed here is not possible with the present data set. However, it is worth noting that stellate cells are more likely to generate spike clusters (i.e., low latency ISIs) in response to depolarizing inputs than pyramidal cells (Alonso and Klink, 1993). Here, we observed that the median probability of observing sub-15 ms ISIs was three times higher in trough locked grid cells than in peak locked grid cells. This suggests that the trough locked grid cells consist, in part at least, of stellate cells.

One implication of the observed phenotypic diversity, with particular relevance for modeling efforts, is that not all phenotypes of grid cell activity must be accounted for in one type of neuron. For example, it may be that the characteristic spatial tuning of grid cells is constructed by one population of cells and that these cells do not exhibit phase precession. Then, the spatial tuning of this first set of neurons is propagated onto another population of cells that, as a result of how the spatial signal is integrated, develops the phase precession phenotype.

Likewise, for empiricists it is important to consider that the magnitude of observed effects will likely differ in a systematic fashion over phenotypically distinct sub-populations of grid cells. As such, analyses should explicitly examine the variance over observations to rule out the possibility of multi-modal distributions of effect sizes. Likewise, when developing and testing hypotheses regarding grid cells, it is important to consider the scope of the hypothesis. That is, if the hypothesis is in regard to how grid tuning arises, not every grid cell may offer an equally valid test of that hypothesis given that the spatial tuning may be generated by only a subset of grid cells and “inherited” by the rest.

### DISSOCIATING GRID CELL PROPERTIES AS A FUNCTION OF PHASE LOCKING PREFERENCE

In the analyses described here, the phase of theta during which individual grid cells were most likely to fire served as a practical and informative covariate by which to assess grid cell properties. The practicality derives from the fact that the local field potential, and thereby the alignment between the spike timing and theta phase, is readily collected in any situation in which grid cell activity is extracellularly recorded. Further, among all of the properties of grid cell spiking considered, none were as strongly bi-modally distributed as the phase of preferred firing, nor did they account for variance among the remaining properties as well.

The differences between trough and peak locked grid cells described here offer new insights into the neural information processing that occurs during these two phases of theta. Processing performed during the trough and peak of theta is hypothesized to support mnemonic retrieval and encoding, respectively (Hasselmo et al., 2002; Douchamps et al., 2013). Here, we found that the spatial information carried in the spiking of trough locked grid cells was substantially greater than that of the peak locked grid cells. The improved spatial tuning of the trough locked grid cells is consistent with an account wherein mnemonic retrieval serves to improve the spatial tuning. Further, the observation that phase precession is observed in trough locked grid cells suggests that precession is a product of retrieval related processing, consistent with previous modeling work (Jensen and Lisman, 1996; Tsodyks et al., 1996; Wallenstein and Hasselmo, 1997; Hasselmo and Eichenbaum, 2005; Norman et al., 2006, 2007).

Theta phase locking preferences have the additional feature of serving as a good predictor of the layer of entorhinal cortex that a cell may be located (Mizuseki et al., 2009). Based on this work, it is likely that the majority of the trough locked grid cells examined here were layer II cells and that the peak locked cells were located in layer III. This is consistent with our observation that the trough locked grid cells were more likely to exhibit significant phase precession, as phase precession has been previously shown be more commonly observed among grid cells in layer II than in layer III (Hafting et al., 2008; Climer et al., 2013).

Notably, however, we found that head direction tuning was also a property of trough locked grid cells. This is at odds with previous work demonstrating that head direction tuning much more likely to be observed in layer III than in layer II (Sargolini et al., 2006; Giocomo et al., 2014). Closer examination of the phase locking preferences of layer III cells reported by Mizuseki et al. (2009; **Figure 3C**), however, reveals that there is a relatively small population of layer III cells that are strongly phase locked to the trough of theta despite the overwhelming majority of the layer III cells are modestly locked to the peak (resembling the peak locked cells analyzed here). Thus, we conclude that the conjunctive cells comprise a sub-population of the layer III cells that fire asynchronously with the remainder of the layer III cells.

The asynchrony between the conjunctive grid cells and the other layer III cells is compelling, in that it violates the standard temporal relationship between the activation of the entorhinal input to the hippocampus and the activation of hippocampal neurons. That is, in the case of entorhinal layer II input to the DG and area CA3 and in the case of entorhinal layer III input to area CA1, the entorhinal input arrives about 180^∘^ out of phase with the activity of the respective hippocampal target areas. In contrast, the sub-population of layer III grid cells with significant head-direction tuning fire immediately prior to when cells in area CA1, to which they putatively project, are most likely to fire. In the context of the hypothesis that this trough locked activity is relates to retrieval processing, it may be that this input serves as a form of a retrieval cue, seeding the activation of area CA1.

### RELATION TO EXISTING COMPUTATIONAL MODELS OF GRID CELLS

While it remains unclear how grid cells derive the characteristic grid tuning, several prominent hypotheses exist in the form of computational models (Burgess et al., 2005, 2007; Fuhs and Touretzky, 2006; Blair et al., 2008; Burgess, 2008; Hasselmo, 2008; Burak and Fiete, 2009; Welday et al., 2011; Grossberg and Pilly, 2012; Hasselmo and Brandon, 2012; Navratilova et al., 2012; Bush and Burgess, 2014; Hasselmo, 2014; Onslow et al., 2014). Few of these models, however, take into consideration that grid cells in layers II and III fire asynchronously. In a recent model from our lab (Hasselmo, 2014), grid cells fire on alternate cycles of a carrier frequency in the course of passing a packet of activity between two populations of grid cells. This mechanism serves to preserve a working memory of the current location of the animal without requiring individual cells to remain tonically active. The original version of this model (Hasselmo, 2014) suggested that the carrier frequency may be the theta rhythm, thereby explaining theta cycle skipping observed in the medial entorhinal cortex (Brandon et al., 2013). The present data, however, suggest that the time scale of the carrier frequency may be twice that of theta, resulting in separate populations of grid cells firing on opposite phases of theta and individual grid cells that fire on a specific phase within each theta cycle while in-field.

### SUMMARY

We have presented data demonstrating that the functional and basic spiking properties of grid cells differ as a function of the phase of theta at which the cells are most likely to fire. The results demonstrate that trough locked grid cells exhibit greater spatial tuning and are more likely to exhibit significant phase precession and head direction tuning. As such, the improved spatial tuning and phase precession may reflect the influence of the retrieval dynamics. The conjunctive grid cells, however, may serve as a retrieval cue, activating 180^∘^ out of phase with the majority of the other layer III neurons.

## Conflict of Interest Statement

The authors declare that the research was conducted in the absence of any commercial or financial relationships that could be construed as a potential conflict of interest.

## References

[B1] Alonso A. Klink R. (1993). Differential electroresponsiveness of stellate and pyramidal-like cells of medial entorhinal cortex layer II. *J. Neurophysiol.* 70 128–143 8395571 10.1152/jn.1993.70.1.128

[B2] Amaral D. G. Witter M. P. (1989). The three-dimensional organization of the hippocampal formation: a review of anatomical data. *Neuroscience* 31 571–591 10.1016/0306-4522(89)90424-7 2687721

[B3] Blair H. T. Gupta K. Zhang K. (2008). Conversion of a phase- to a rate-coded position signal by a three-stage model of theta cells, grid cells, and place cells. *Hippocampus* 18 1239–1255 10.1002/hipo.20509 19021259 PMC2814603

[B4] Brandon M. P. Bogaard A. R. Libby C. P. Connerney M. A. Gupta K. Hasselmo M. E. (2011). Reduction of theta rhythm dissociates grid cell spatial periodicity from directional tuning. *Science* 332 595–599 10.1126/science.1201652 21527714 PMC3252766

[B5] Brandon M. P. Bogaard A. R. Schultheiss N. W. Hasselmo M. E. (2013). Segregation of cortical head direction cell assemblies on alternating 𝜃 cycles. *Nat. Neurosci.* 16 739–748 10.1038/nn.3383 23603709 PMC3703458

[B6] Brankack J. Stewart M. Fox S. E. (1993). Current source density analysis of the hippocampal theta rhythm: associated sustained potentials and candidate synaptic generators. *Brain Res.* 615 310–327 10.1016/0006-8993(93)90043-M 8364740

[B7] Burak Y. Fiete I. R. (2009). Accurate path integration in continuous attractor network models of grid cells. *PLoS Comput. Biol.* 5:e1000291 10.1371/journal.pcbi.1000291 PMC2632741 19229307

[B8] Burgess N. (2008). Grid cells and theta as oscillatory interference: theory and predictions. *Hippocampus* 18 1157–1174 10.1002/hipo.20518 19021256 PMC3196519

[B9] Burgess N. Barry C. Jeffery K. J. O’Keefe J. (2005). “A grid and place cell model of path integration utilizing phase precession versus theta,” in *Proceedings of the 1st Annual Conference of Computational Cognitive Neuroscience, November* 10–11 Washington, DC

[B10] Burgess N. Barry C. O’Keefe J. (2007). An oscillatory interference model of grid cell firing. *Hippocampus* 17 801–812 10.1002/hipo.20327 17598147 PMC2678278

[B11] Bush D. Burgess N. (2014). A hybrid oscillatory interference/continuous attractor network model of grid cell firing. *J. Neurosci.* 34 5065–5079 10.1523/JNEUROSCI.4017-13.2014 24695724 PMC3972729

[B12] Buzsáki G. Leung L. W. Vanderwolf C. H. (1983). Cellular bases of hippocampal EEG in the behaving rat. *Brain Res.* 287 139–171 10.1016/0165-0173(83)90037-1 6357356

[B13] Canto C. B. Witter M. P. (2012). Cellular properties of principal neurons in the rat entorhinal cortex. II. The medial entorhinal cortex. *Hippocampus* 22 1277–1299 10.1002/hipo.20993 22161956

[B14] Canto C. B. Wouterlood F. G. Witter M. P. (2008). What does the anatomical organization of the entorhinal cortex tell us? *Neural Plast*. 2008 381243 10.1155/2008/381243 PMC2526269 18769556

[B15] Climer J. R. Newman E. L. Hasselmo M. E. (2013). Phase coding by grid cells in unconstrained environments: two-dimensional phase precession. *Eur. J. Neurosci.* 38 2526–2541 10.1111/ejn.12256 23718553 PMC3912569

[B16] Deshmukh S. S. Knierim J. J. (2011). Representation of non-spatial and spatial information in the lateral entorhinal cortex. *Front. Behav. Neurosci.* 5:69 10.3389/fnbeh.2011.00069 PMC3203372 22065409

[B17] Domnisoru C. Kinkhabwala A. A. Tank D. W. (2013). Membrane potential dynamics of grid cells. *Nature* 495 199–204 10.1038/nature11973 23395984 PMC4099005

[B18] Douchamps V. Jeewajee A. Blundell P. Burgess N. Lever C. (2013). Evidence for encoding versus retrieval scheduling in the hippocampus by theta phase and acetylcholine. *J. Neurosci.* 33 8689–8704 10.1523/JNEUROSCI.4483-12.2013 23678113 PMC3715394

[B19] Dragoi G. Buzsáki G. (2006). Temporal encoding of place sequences by hippocampal cell assemblies. *Neuron* 50 145–157 10.1016/j.neuron.2006.02.023 16600862

[B20] Fuhs M. C. Touretzky D. S. (2006). A spin glass model of path integration in rat medial entorhinal cortex. *J. Neurosci.* 26 4266–4276 10.1523/JNEUROSCI.4353-05.2006 16624947 PMC6674007

[B21] Fyhn M. Molden S. Witter M. P. Moser E. I. Moser M.-B. (2004). Spatial representation in the entorhinal cortex. *Science* 305 1258–1264 10.1126/science.1099901 15333832

[B22] Giocomo L. M. Stensola T. Bonnevie T. Van Cauter T. Moser M.-B. Moser E. I. (2014). Topography of head direction cells in medial entorhinal cortex. *Curr. Biol.* 24 252–262 10.1016/j.cub.2013.12.002 24440398

[B23] Grossberg S. Pilly P. K. (2012). How entorhinal grid cells may learn multiple spatial scales from a dorsoventral gradient of cell response rates in a self-organizing map. *PLoS Comput. Biol.* 8:e1002648 10.1371/journal.pcbi.1002648 PMC3464193 23055909

[B24] Hafting T. Fyhn M. Bonnevie T. Moser M.-B. Moser E. I. (2008). Hippocampus-independent phase precession in entorhinal grid cells. *Nature* 453 1248–1252 10.1038/nature06957 18480753

[B25] Hafting T. Fyhn M. Molden S. Moser M.-B. Moser E. I. (2005). Microstructure of a spatial map in the entorhinal cortex. *Nature* 436 801–806 10.1038/nature03721 15965463

[B26] Hasselmo M. E. (2008). Grid cell mechanisms and function: contributions of entorhinal persistent spiking and phase resetting. *Hippocampus* 18 1213–1229 10.1002/hipo.20512 19021258 PMC2614862

[B27] Hasselmo M. E. (2014). Neuronal rebound spiking, resonance frequency and theta cycle skipping may contribute to grid cell firing in medial entorhinal cortex. *Philos. Trans. R. Soc. Lond. B Biol. Sci.* 369 20120523 10.1098/rstb.2012.0523 PMC3866445 24366135

[B28] Hasselmo M. E. Bodelón C. Wyble B. P. (2002). A proposed function for hippocampal theta rhythm: separate phases of encoding and retrieval enhance reversal of prior learning. *Neural Comput.* 14 793–817 10.1162/089976602317318965 11936962

[B29] Hasselmo M. E. Brandon M. P. (2012). A model combining oscillations and attractor dynamics for generation of grid cell firing. *Front. Neural Circuits* 6:30 10.3389/fncir.2012.00030 PMC3361022 22654735

[B30] Hasselmo M. E. Eichenbaum H. (2005). Hippocampal mechanisms for the context-dependent retrieval of episodes. *Neural Netw.* 18 1172–1190 10.1016/j.neunet.2005.08.007 16263240 PMC2253492

[B31] Jensen O. Lisman J. E. (1996). Hippocampal CA3 region predicts memory sequences: accounting for the phase precession of place cells. *Learn. Mem.* 3 279–287 10.1101/lm.3.2-3.279 10456097

[B32] Kempter R. Leibold C. Buzsáki G. Diba K. Schmidt R. (2012). Quantifying circular-linear associations: hippocampal phase precession. *J. Neurosci. Methods* 207 113–124 10.1016/j.jneumeth.2012.03.007 22487609

[B33] Kitamura T. Pignatelli M. Suh J. Kohara K. Yoshiki A. Abe K. (2014). Island cells control temporal association memory. *Science* 343 896–901 10.1126/science.1244634 24457215 PMC5572219

[B34] Kubie J. L. Muller R. U. Bostock E. (1990). Spatial firing properties of hippocampal theta cells. *J. Neurosci.* 10 1110–1123 2329371 10.1523/JNEUROSCI.10-04-01110.1990 PMC6570223

[B35] Mitchell S. J. Ranck J. B. (1980). Generation of theta rhythm in medial entorhinal cortex of freely moving rats. *Brain Res.* 189 49–66 10.1016/0006-8993(80)90006-2 7363097

[B36] Mizuseki K. Sirota A. Pastalkova E. Buzsáki G. (2009). Theta oscillations provide temporal windows for local circuit computation in the entorhinal-hippocampal loop. *Neuron* 64 267–280 10.1016/j.neuron.2009.08.037 19874793 PMC2771122

[B37] Navratilova Z. Giocomo L. M. Fellous J.-M. Hasselmo M. E. McNaughton B. L. (2012). Phase precession and variable spatial scaling in a periodic attractor map model of medial entorhinal grid cells with realistic after-spike dynamics. *Hippocampus* 22 772–789 10.1002/hipo.20939 21484936

[B38] Newman E. L. Climer J. R. Hasselmo M. E. (2014). Grid cell spatial tuning reduced following systemic muscarinic receptor blockade. *Hippocampus* 24 643–655 10.1002/hipo.22253 24493379 PMC4028397

[B39] Newman E. L. Gillet S. N. Climer J. R. Hasselmo M. E. (2013). Cholinergic blockade reduces theta-gamma phase amplitude coupling and speed modulation of theta frequency consistent with behavioral effects on encoding. *J. Neurosci.* 33 19635–19646 10.1523/JNEUROSCI.2586-13.2013 24336727 PMC3858632

[B40] Norman K. A. Newman E. L. Detre G. (2007). A neural network model of retrieval-induced forgetting. *Psychol. Rev.* 114 887–953 10.1037/0033-295X.114.4.887 17907868

[B41] Norman K. A. Newman E. L. Detre G. J. Polyn S. M. (2006). How inhibitory oscillations can train neural networks and punish competitors. *Neural Comput.* 18 1577–1610 10.1162/neco.2006.18.7.1577 16764515

[B42] Onslow A. C. E. Hasselmo M. E. Newman E. L. (2014). DC-shifts in amplitude in-field generated by an oscillatory interference model of grid cell firing. *Front. Syst. Neurosci.* 8:1 10.3389/fnsys.2014.00001 PMC3901010 24478639

[B43] Sargolini F. Fyhn M. Hafting T. McNaughton B. L. Witter M. P. Moser M.-B. (2006). Conjunctive representation of position, direction, and velocity in entorhinal cortex. *Science* 312 758–762 10.1126/science.1125572 16675704

[B44] Schmitzer-Torbert N. Jackson J. Henze D. Harris K. Redish A. D. (2005). Quantitative measures of cluster quality for use in extracellular recordings. *Neuroscience* 131 1–11 10.1016/j.neuroscience.2004.09.066 15680687

[B45] Sharp P. E. Koester K. (2008). Lesions of the mammillary body region alter hippocampal movement signals and theta frequency: implications for path integration models. *Hippocampus* 18 862–878 10.1002/hipo.20474 18702112

[B46] Skaggs W. E. McNaughton B. L. Gothard K. M. Markus E. J. (1993). An information-theoretic approach to deciphering the hippocampal code. *Neural Inf. Process Syst.* 5 1030–1037

[B47] Solstad T. Boccara C. N. Kropff E. Moser M.-B. Moser E. I. (2008). Representation of geometric borders in the entorhinal cortex. *Science* 322 1865–1868 10.1126/science.1166466 19095945

[B48] Steward O. (1976). Topographic organization of the projections from the entorhinal area to the hippocampal formation of the rat. *J. Comp. Neurol.* 167 285–314 10.1002/cne.901670303 1270625

[B49] Steward O. Scoville S. A. (1976). Cells of origin of entorhinal cortical afferents to the hippocampus and fascia dentata of the rat. *J. Comp. Neurol.* 169 347–370 10.1002/cne.901690306 972204

[B50] Tsodyks M. V. Skaggs W. E. Sejnowski T. J. McNaughton B. L. (1996). Population dynamics and theta rhythm phase precession of hippocampal place cell firing: a spiking neuron model. *Hippocampus* 6 271–280 10.1002/(SICI)1098-1063(1996)6:3<271::AID-HIPO5>3.0.CO;2-Q 8841826

[B51] Wallenstein G. V. Hasselmo M. E. (1997). Functional transitions between epileptiform-like activity and associative memory in hippocampal region CA3. *Brain Res. Bull.* 43 485–493 10.1016/S0361-9230(97)00003-8 9250622

[B52] Welday A. C. Shlifer I. G. Bloom M. L. Zhang K. Blair H. T. (2011). Cosine directional tuning of theta cell burst frequencies: evidence for spatial coding by oscillatory interference. *J. Neurosci.* 31 16157–16176 10.1523/JNEUROSCI.0712-11.2011 22072668 PMC3758572

[B53] Zhang S.-J. Ye J. Miao C. Tsao A. Cerniauskas I. Ledergerber D. (2013). Optogenetic dissection of entorhinal-hippocampal functional connectivity. *Science* 340 1232627 10.1126/science.1232627 23559255

